# A Cross-Sectional Study to Evaluate the Impact of Placental Location on Maternal and Fetal Outcomes

**DOI:** 10.7759/cureus.40291

**Published:** 2023-06-12

**Authors:** Naveena Alakonda, Neelamma Patil, Rajasri Yaliwal, Aruna Biradar, Shoba Shiragur, Shreedevi Kori, Sangamesh Mathapati

**Affiliations:** 1 Obstetrics and Gynaecology, Shri B. M. Patil Medical College, Vijayapura, IND

**Keywords:** preeclampsia, prom, hpr, left lateral, fundal, placental location

## Abstract

Background

The human placenta is a critical organ for facilitating nutrient uptake, waste elimination, and gaseous exchange between the mother and the fetus. In the placenta, there are two circulations, namely, maternal and fetal. The blood supply of the placenta is not uniformly distributed as the maternal circulation, determined by uterine blood supply, depends on the implantation site. The uterine blood supply plays a significant role in placental blood flow and pregnancy success. Hence, abnormalities in the placental implantation may affect the placenta’s blood supply, leading to adverse maternal and fetal outcomes. This study aims to examine the relationship between placental location and maternofetal outcomes.

Methodology

This cross-sectional study was conducted in the Department of Obstetrics & Gynaecology at Bijapur Lingayat District Educational Association (Deemed to be University), Shri B. M. Patil Medical College, Hospital and Research Centre, Vijayapura, from January 2021 to April 2022. A total of 1,301 patients were included in this study.

Results

There was a positive and significant association between fundal implantation and severe preeclampsia and premature rupture of membranes. There was a positive and significant association between left lateral implantation and severe preeclampsia. The histopathological changes seen in the histopathological report of the fetal surface of the placenta also had a positive and significant association with the fundal location of the placenta.

Conclusions

This study suggests that fundal left lateral placentation leads to abnormal results. Such patients can be considered high risk and should be given meticulous antenatal care, depending on placental location at the 28-week ultrasonography.

## Introduction

The human placenta serves as a crucial conduit for metabolic exchange, endocrine function, and other bodily processes between the mother and the unborn. Additionally, it is a crucial organ that joins the fetus to the uterine wall [1]. Maternal and fetal circulations exist in the placenta. The maternal circulation of the placenta depends on the site of implantation and the resulting placement of the placenta within the uterus; therefore, the placenta’s blood supply is not distributed evenly. Therefore, the uterine blood supply plays a significant role in the success of placental blood flow and pregnancy [2]. Hence, abnormalities in the placental implantation may affect the blood supply of the placenta, leading to adverse maternal and fetal outcomes, such as gestational hypertension, preeclampsia, gestational diabetes, malpresentation, malposition, preterm birth, small for gestational age, fetal growth retardation, low birth weight, intrauterine fetal death, and stillbirth [3].

Numerous branches from each of the two uterine arteries nourish the respective side of the uterus [4]. Both uterine arteries in patients with centrally placed placentas show comparable resistance, and the uteroplacental blood flow requirements are satisfied by equal distribution from both uterine arteries, which is absent when the placenta is laterally positioned [5].

Preeclampsia, fetal distress during birth, abdominal pain, abdominal deliveries, and fetal growth retardation have all been associated with unilateral placental implantations [6,7]. Cornual implantations have been linked with an increased risk of breech presentations [8], persistent placenta previa with fetal growth restriction, abruptio placenta, and third-trimester bleeding [9].

The American College of Obstetricians and Gynecologists and the American Institute of Ultrasound in Medicine recommend that the standard obstetric sonogram in the second and third trimesters should evaluate placental position and morphology [10,11].

Hence, in this study, we evaluated the relationship between placental location and pregnancy outcomes based on placental location at the 28-week ultrasonography.

## Materials and methods

Data source

This study included patients who were admitted to Shri B. M. Patil Medical College, Hospital and Research Centre in Bijapur Lingayat District Educational Association (Deemed to be University), Vijayapura.

Inclusion and exclusion criteria

We included all patients with singleton pregnancies of gestational age >28 weeks who provided informed and written consent. Those with gestational age <28 weeks, multiple pregnancies, uterine anomalies, and who did not give informed and written consent were excluded.

Sample size calculation

With the anticipated proportion of placental insufficiency in the abnormally located placenta at 2.5% [10], the study required a sample size of 1,301 patients with a 99% level of confidence and 1% absolute precision. The following formula was used for sample size calculation: n = z2 p*q/d2, where Z is the Z statistic at α level of significance, d2 is the absolute error, P is the proportion rate, and q is 100-p.

Methodology

For all patients enrolled in our study, ultrasonography was done to look for placenta location on admission. Maternal outcomes including mode of delivery; gravid status when the patient was admitted for delivery; complications such as pregnancy-induced hypertension, abruptio placenta, fetal growth restriction, oligohydramnios, polyhydramnios, premature rupture of membranes, and preterm delivery; and perinatal outcomes such as birth weight, APGAR score, respiratory distress syndrome, hypoxic-ischemic encephalopathy, neonatal intensive care unit admission, intrauterine death, and neonatal death were noted. The placenta was sent for histopathology.

Statistical analysis

The collected data were entered into a Microsoft Excel sheet, and SPSS (IBM Corp., Armonk, NY, USA) was used to conduct statistical analysis. Results are presented as mean (median) ±SD, counts and percentages, and diagrams. Normally distributed continuous variables were compared using the independent t-test. For non-normally distributed variables, the Mann-Whitney U test was used. Categorical variables were compared using the chi-square test. P-values <0.05 were considered statistically significant. All statistical tests two-tailed.

## Results

In our study, 1,861 cases were screened, 250 patients were excluded as they did not meet the inclusion criteria, 88 patients did not consent to participate, and 222 patients were excluded as their placentas could not be sent for histopathological analysis. A total of 1,301 cases were included in the study. Among all the patients, 36.8% patients had an anterior location of the placenta, 22.2% had a fundal location, 3.6% had a left lateral area of the placenta, 30% had a posterior location, 5.1% had a right lateral location, and 2.3% of patients had placenta previa. Most patients belonged to the age group of 21-30 years, while the majority of patients included in the study were multigravida. The maximum number of patients underwent LSCS (Table 1). Blood grouping and Rh typing distribution of our research were as follows: O positive was the most common, followed by B positive, A positive, O negative, AB positive, B negative, A negative, and AB negative blood group being the minor blood group in our study. Various changes were seen on the maternal surface, fetal surface, and decidual part of the placenta on histopathological reporting.

**Table 1:** Relationship between placental location and maternal characteristics. RL = right lateral; LL = left lateral; PP = placenta previa; LSCS = lower-segment cesarean section

Parameter	Fundal	RL	LL	Posterior	Anterior	PP	P-value
Age (21–30 years) (%)	21.1%	4.9%	4.4%	30.8%	36.6%	2.3%	0.0116
Multigravida (%)	23.2%	5.4%	3.4%	29.2%	36.4%	2.3%	0.679
LSCS (%)	57.9%	43.9%	49%	62.8%	61.6%	70%	0.0382

There was a positive and significant association between the fundal location of the placenta and PROM (p = 0.038) (Table 2), as well as between the fundal and severe pre-eclampsia (p = 0.006) (Table 3). A positive and significant association was seen between left lateral and severe PE (p = 0.043) (Table 4).

**Table 2:** Association between the fundal location of the placenta and PROM. *: statistically significant. PROM = premature rupture of membranes

			Fundus		Total	Chi-square value	P-value
			No	Yes			
PROM	No	Count	946	261	1,207		
		% within fundus	93.6%	90.0%	92.8%		
	Yes	Count	65	29	94	4.287	0.038*
		% within fundus	6.4%	10.0%	7.2%		
Total		Count	1,011	290	1,301		
		% within fundus	100.0%	100.0%	100.0%		

**Table 3:** Association between the fundal location of the placenta and severe PE. *: statistically significant. PE = preeclampsia

			Fundus		Total	Chi-square value	P-value
			No	Yes			
Severe PE	No	Count	914	277	1,191		
		% within fundus	90.4%	95.5%	91.5%		
	Yes	Count	97	13	110	7.608	0.006*
		% within fundus	9.6%	4.5%	8.5%		
Total		Count	1,011	290	1,301		
		% within fundus	100.0%	100.0%	100.0%		

**Table 4:** Association between the left lateral placenta and severe PE. *: statistically significant. PE = preeclampsia

			Left lateral		Total	Chi-square value	P-value
			No	Yes			
Severe PE	No	Count	1,150	41	1,191		
		% within LL	91.9%	83.7%	91.5%		
	Yes	Count	102	8	110	4.076	0.043*
		% within LL	8.1%	16.3%	8.5%		
Total		Count	1,252	49	1,301		
		% within LL	100.0%	100.0%	100.0%		

However, a positive and significant association was seen between fundal implantation of the placenta and changes seen on the fetal surface of the placenta on histopathological reporting (p = 0.030) (Table 5). No significant association was seen between various placental locations and fetal outcomes (Table 6).

**Table 5:** Association between the fundal location of the placenta and the fetal surface of the placenta. *: statistically significant.

			Fundus		Total	Chi-square value	P-value
			No	Yes			
Fetal surface	Chorangiosis	Count	16	3	19		
		% Fundus	1.6%	1.0%	1.5%		
	Clustered villi	Count	391	125	516		
		% Fundus	38.7%	43.1%	39.7%		
	Clustered villi, chorangiosis	Count	275	60	335		
		% Fundus	27.2%	20.7%	25.7%	10.701	0.030*
	Clustered villi, small thrombi in blood vessels	Count	6	6	12		
		% Fundus	0.6%	2.1%	0.9%		
	Normal	Count	323	96	419		
		% Fundus	31.9%	33.1%	32.2%		
Total		Count	1,011	290	1,301		
		% Fundus	100.0%	100.0%	100.0%		

**Table 6:** Association between the placental location and fetal outcomes. NICU = neonatal intensive care unit; MS = mother’s side; FSB = fresh stillborn; MSB = macerated stillborn

Fetal outcome	Anterior	Fundal	LL	Posterior	PP	RL	P-value
NICU	54.7%	49.1%	42.6%	52,3%	70%	53%	0.16
MS	42%	46.4%	51.1%	43.1%	30%	42.4%	0.44
FSB	2.7%	2.8%	2.1%	3.8%	0%	4.5%	0.75
MSB	0.2%	0.7%	2.1%	0.5%	0%	0%	0.50

## Discussion

There is a dearth of information on the relationship between placental position, perinatal outcomes, and pregnancy problems. Few studies have discussed how the position of the placenta affects pregnancy complications such as preterm birth, pre-eclampsia, and small for gestational age [12,13].

In this study, we discovered that, when compared to other placenta placements, the anterior position is the most typical. Numerous other authors have made similar claims, including Pai et al. [4], Patel et al. [14], Liberti et al. [15], Bhalerao et al. [16], Magann et al. [17], and Kartika Devrajan et al. [18]. However, numerous additional investigations, including those by Kofinas et al. [19], Liberti et al. [15], Kakkar, and Contro et al. [20] discovered that the lateral placenta was more typical. These variations can result from different standards for classifying the central and lateral placentas. The standards used in this investigation and the study by Bhalerao et al. [16] were the same, and the findings were also consistent.

In this study, there was a positive and significant association between fundal implantation of the placenta and severe PE and PROM. These results are consistent with those of another study [21]. Hadley et al. [12] have provided a potential justification, stating that placental fundal implantation places the membrane’s weakest point over the cervical OS. Thus, it raises the possibility of developing premature membrane rupture.

There was a positive and significant association between the left lateral position and severe PE. These statistically significant correlations were in line with the study by Kakkar et al. [22], who examined 150 women and found that 70% of those with lateral placentas went on to develop PE. Pai et al. [4] examined 426 pregnancies, of which 71 had PE and 74% had a placenta that was laterally positioned.

## Conclusions

The implantation of the placenta determined at 28 weeks can be used to evaluate pregnancies and categorize them into high-risk groups for maternal complications and fetal outcomes. Severe PE and PROM are significantly associated with the location of the fundal placenta. The left lateral location of the placenta has a significant association with severe PE. The placement of the placenta, as identified by USG, can be utilized as a non-invasive, inexpensive, and simple predictor of unfavorable pregnancy outcomes. The advantage of USG is that it is a part of routine antenatal care, so there is no extra cost. Therefore, this study complements the hypothesis that placental location and pregnancy outcomes are interlinked. However, this observation needs additional research to confirm the findings.
